# The transition to Teletherapy: Experiences of emotionally focused therapists

**DOI:** 10.1111/famp.13068

**Published:** 2024-10-14

**Authors:** Caitlin Edwards, Andrea K. Wittenborn, Preston Morgan, Francesca Pratt, Katie Heiden‐Rootes

**Affiliations:** ^1^ Human Development and Family Studies Colorado State University Fort Collins Colorado USA; ^2^ Human Development and Family Studies Michigan State University East Lansing Michigan USA; ^3^ Psychiatry and Behavioral Medicine Michigan State University Grand Rapids Michigan USA; ^4^ Human Development and Family Science Oklahoma State University Stillwater Oklahoma USA; ^5^ Family and Community Medicine Saint Louis University Saint Louis Missouri USA

**Keywords:** COVID‐19 pandemic, emotionally focused therapy, teletherapy

## Abstract

Emotionally focused therapy (EFT) is an evidence‐based treatment for relational distress based on experiential, humanistic, and attachment theories. Despite the empirical support for EFT, there are no studies on EFT delivered via teletherapy. In this study, we aimed to understand therapists' experiences delivering EFT through teletherapy using open‐ended questions on a web‐based survey of certified EFT therapists (*n* = 69). Reflexive thematic analysis identified five themes: (1) Delivering EFT via teletherapy is similar to in‐person therapy, (2) Delivering EFT via teletherapy is more challenging than in‐person therapy, (3) Delivering EFT via teletherapy is more challenging with certain clients, (4) therapists adapted EFT for teletherapy, and (5) teletherapy and the COVID‐19 pandemic changed therapy practice. The themes illustrated the mixed experiences of EFT therapists using teletherapy. Whereas some found it straightforward to use EFT via teletherapy with couples, others experienced exhaustion and barriers. Practice recommendations for delivering EFT via teletherapy are outlined, including modifying assessment strategies, implementing new safety protocols, and providing additional psychoeducation.

The global COVID‐19 pandemic and its associated mortality, stay‐at‐home orders, and novel external stressors resulted in a significant demand for relationship and mental health clinicians to provide therapy online. While couple and family therapists (CFTs) may be uniquely suited to help clients struggling relationally during times of high stress (Hardy et al., 2021; Lebow, 2020; Stanley & Markman, 2020), Blumer et al. (2015) reported CFTs felt unprepared to conduct teletherapy. Allan et al. (2021), Brenner (2021), Hogan (2022), O'Reilly Treter et al. (2021), and Geller (2021) have provided conceptual discussions and recommendations for teletherapy; however, there is limited research on how therapists deliver CFT via teletherapy. While Blackhaus et al. (2012) conducted a systematic review assessing the effectiveness of individual teletherapy, no systematic reviews and few studies have been conducted on teletherapy for couples. Research on couple teletherapy has primarily focused on the experience of therapists transitioning to teletherapy (Hardy et al., 2021), building teletherapeutic alliance (Aviram & Nadan, 2023; Glass & Bickler, 2021), and therapist attitudes toward teletherapy (Machluf et al., 2021).

Doss et al. (2013) and Doss and Hatch (2022) provide a unique description of translating Integrative Behavioral Couple Therapy (IBCT; Jacobson & Christensen, 1996), a behavioral evidence‐based model of couple therapy, to an online psychoeducation format. IBCT and EFT are the only well‐established therapeutic models for treating romantic relationship distress, making the translation of IBCT to the online OurRelationship program especially relevant. The OurRelationship program (Doss et al., 2013) consists of seven to 10 h of online content to identify relationship problems, understand those problems in more depth, and develop solutions for those problems supplemented by brief coaching via telephone by a therapist (Doss et al., 2013). Although the OurRelationship program has been shown to improve relationship functioning (Doss et al., 2016, 2020; Hatch et al., 2022; Roddy et al., 2018), the program primarily focuses on creating behavior‐based changes as opposed to changes rooted in emotional experiencing, emotional coherence, and attachment security (Doss et al., 2013). Doss and Hatch (2022) report that it is challenging to elicit more vulnerable emotions online and the OurRelationship program has compensated by providing scripted coaching calls with a therapist. This calls into question how well affective and experiential‐based empirically supported treatments, such as emotionally focused therapy (EFT) can be translated to an online format. Thus, this study sought to explore EFT therapists' experiences delivering EFT through teletherapy following the stay‐at‐home orders during the COVID‐19 pandemic.

## Emotionally focused therapy

EFT is an experiential, present‐focused, empirically supported treatment for relational distress. In EFT, partners are guided through moment‐to‐moment experiencing by an accurately attuned therapist with the purpose of fostering a secure attachment bond. Therapists track and reflect partners' emotional experiencing, creating deep emotional engagement, and thereby enable change through vulnerable enactments and experiences of co‐regulation. EFT is organized into three stages, the first two demarcated by the level of emotional engagement between partners. Stage one involves the de‐escalation of negative client interaction patterns, resulting in an experiential understanding of the negative cycle and each partner's attachment fears. Stage two includes two significant change events, withdrawer re‐engagement and pursuer softening, in which each partner experientially encounters attachment fears and needs and shares these with their partner. The goal is to increase attachment security through emotional experiencing that enables the couple to ask and receive contact, care, and comfort in times of distress (Johnson, 2004; 2019). Stage three involves consolidation of change.

### Emotionally focused therapy online

Due to the highly experiential nature of EFT, there are questions as to whether and how EFT will align with service delivery via teletherapy. Specifically, the literature has not yet examined the use of EFT via teletherapy for the development and maintenance of therapeutic alliance, tracking and reflecting client micro‐expressions, heightening and deepening client emotional experience, and enactments. Regarding the therapeutic alliance, while clients rate therapeutic alliance over teletherapy and in person similarly, therapists often rate therapeutic alliance higher in person compared to teletherapy (Lopez et al., 2019). The scarce literature on CFT delivered via teletherapy suggests that developing a teletherapeutic alliance is facilitated by therapists being highly attuned to auditory, visual, verbal, and non‐verbal responses, as well as clients' tone (Glass & Bickler, 2021; Springer et al., 2020).

The need to be highly attuned to minimal cues parallels the emphasis of EFT on micro‐expressions (Johnson, 2019). Micro‐expressions are fleeting signs of concealed emotions (Ekman, 2003) that are often outside of awareness and are emphasized in EFT as a pathway to deeper emotional coherence and engagement (Johnson, 2019). Micro‐expressions involve both the face and the body (Ekman, 2003). As working online inherently involves a narrowing of physical space, therapists may not be able to gauge a client's full body or facial expression and may be more likely to miss salient emotional moments. Glass and Bickler (2021) discuss the need for therapists to be more verbally explicit when working via teletherapy due to the lack of physical presence and attunement.

Several interventions in EFT, such as heightening and enactments, could pose challenges for therapists when delivering services online due to special and technological limitations. For example, the intervention of heightening in EFT serves the important purpose of drawing attention to and amplifying a client's emotional experiencing (Johnson, 2019). Allan et al. (2020) discuss heightening the use of client words and phrases to engage clients in vivid emotional experiencing online. In person, heightening often involves the use of evocative imagery and repetition using simple words, a slow pace, and a soft voice (Johnson, 2019). It is possible that heightening may be impacted by the constraints of technology and the need to be louder and more verbally explicit about the purpose of therapeutic interventions.

Enactments (i.e., engaged encounters) in EFT involve sharing a concrete and coherent deepened emotional reality (Johnson, 2019). An EFT therapist will often ask one partner to turn to the other and vulnerably share their newly coherent emotional experience (Johnson, 2019). This is an essential ingredient in EFT, as couples learn to co‐regulate and communicate more authentically through engaged encounters (Johnson, 2019). However, the limitations of therapeutic space, the potential for escalation between couples, and the barriers of technology may make these enactments less feasible. Allan et al. (2020) suggest therapists ensure this disclosure is direct and can be received without distraction or escalation. If there is a risk for escalation, the therapist should act as a temporary attachment figure and have the client share with the therapist, rather than with their partner (Allan et al., 2020). Other suggestions include displaying empathy, using a consistently soothing tone, and having the partner share a safer aspect of their emotional experience (Allan et al., 2020).

## The current study

Despite the empirical support for EFT, there is a lack of research on implementing EFT via teletherapy. This lack of teletherapy research is not unique to EFT, but common across couple therapy approaches. There was a recent surge in published work on teletherapy in the field of couple therapy, however, the articles were primarily thought pieces (Brenner, 2021; Burgoyne & Cohn, 2020) or qualitative studies on training students in couple therapy (e.g., Heiden‐Rootes et al., 2021; Pickens et al., 2020) or clinicians' general experiences with teletherapy (Eppler, 2021; Hardy et al., 2021). Even in this larger literature, there is a gap in knowledge on how to deliver evidence‐based treatments to couples via teletherapy.

Given the transition to teletherapy during the COVID‐19 pandemic, it is important to understand EFT therapists' experiences of implementing EFT via teletherapy. While Allan et al. (2020) shared valuable clinical recommendations for using EFT via teletherapy, empirical studies are needed to provide more insight into the delivery of EFT for teletherapy. In this study, we aim to use reflexive thematic analysis to better understand EFT therapists' experiences delivering EFT via teletherapy following the stay‐at‐home orders during the COVID‐19 pandemic. Specifically, we aim to explore the research question: *How did EFT therapists experience delivering EFT through teletherapy during the stay‐at‐home orders in the COVID‐19 pandemic?* Capturing the transition from in person to telehealth would allow for a contrast to be made by the participants in real‐time and provide descriptions of skills or competencies that may need to be addressed in EFT training and couple therapy telehealth, more generally.

## METHOD

All study procedures were approved by the Saint Louis University Institutional Review Board (#31185). The study was deemed exempt due to the nature of the survey and data being gathered. Written and verbal consent were waived.

### Procedure

We used criterion sampling (Palinkas et al., 2015) to identify and recruit therapists with expertise in EFT. Potential participants were contacted via email listservs or social media to colleagues and organizations known by the research team. Inclusion criteria included: (1) At least 18 years old; (2) Certified in EFT; (3) Clinically active with at least 5 hours per week of practice with couples; and (4) Moved from an in‐person‐based therapy to teletherapy service due to the COVID‐19 pandemic in 2020. Certification in EFT requires at least a provisional license as a mental health professional in the state or province where clinical practice is occurring. All participants were informed of the study procedures and were provided a recruitment statement prior to participating. Each participant completed an online survey that included demographic and open‐ended questions. After completion of the study, participants were offered the chance to enter their name into a raffle for a $25 Amazon gift card. Data were collected from February 2021 to June 2021.

### Participants

In this study, 69 therapists participated in the qualitative surveys. Fifty‐eight participants identified as female, and 11 participants identified as male. Most participants (88%) were racially White, had earned master of arts or science degrees (*n* = 40), 15 participants earned doctoral degrees, and 14 participants identified earning other graduate degrees (e.g., master's in social work, Psy.D., Ed.D., etc.). Participants held the following licenses: marriage and family therapy (*n* = 26), social work (*n* = 19), psychologist (*n* = 16), professional counselor (*n* = 13), and others (*n* = 3). Most participants lived in the United States (*n* = 63), while six lived in Canada. All participants reported providing video sessions; seven participants provided video and phone sessions.

### Measures

Participants completed a web‐based survey that included questions about participant demographics and open‐ended survey questions on delivering EFT through teletherapy compared to in person. Open‐ended questions were designed to ask about each stage of EFT. Examples of questions include: (a) Describe any differences you have noticed in forming and maintaining an alliance in sessions when using in‐person therapy compared to teletherapy, (b) Describe any differences you have noticed in identifying and tracking the cycle in sessions when using in‐person therapy compared to teletherapy, and (c) Describe any differences you have noticed in accessing underlying emotions and attachment needs when using in‐person therapy compared to teletherapy. Participants were asked to specify whether they were describing delivering EFT through video or phone sessions.

### Analysis

We used reflexive thematic analysis (Braun & Clarke, 2006, 2021) to analyze the open‐ended survey questions. Two scholars with expertise in EFT (i.e., completed all requirements for certification) followed the six‐step process. First, we became familiar with the data by reading participant responses. Second, we each read and coded the data and then met to discuss our codes and compare our interpretations of the data. Third, we reviewed our coded data and generated initial themes. Fourth, we reviewed and developed themes. Fifth, we further refined, defined, and named the themes we identified. Sixth, we prepared the report. We used an inductive approach to code the data and develop themes based in the responses provided by the participants. Further, we used a combination of latent and semantic approaches to code the data (Braun & Clarke, 2021). We used a latent approach to interpret the underlying or hidden meanings of the participants' responses. When the surface meaning of the response was more clear, semantic coding was used to capture the explicit concept shared by participants. Most participants only used video‐conferencing, yet seven (10%) participants also conducted some phone sessions. All data were collected and coded together, but comments were distinguished by technology (i.e., video or phone). In the results, the term teletherapy refers to findings relevant to both video‐conferencing and phone sessions, while findings relevant to either video‐conferencing or phone sessions are clearly labeled.

### Trustworthiness

The trustworthiness of qualitative research is determined by the degree to which the study procedures (e.g., data collection, data analysis) and researcher positions have been made transparent (Adler, 2022; Hadi & Closs, 2016). At the time of data collection, all authors were members of COAMFTE‐accredited graduate degree programs in midwestern universities and identified as White (including afforded privileges). The first and fourth authors both transitioned to providing teletherapy at the same time as research participants, which paralleled many of the experiences detailed by the participants. The data were coded by the first and second authors who have expertise in EFT delivery (i.e., completed all requirements for certification) and scholarship (i.e., published peer‐reviewed conceptual and empirical articles on EFT). The coders met to discuss the data, coding, and themes. Per Braun and Clarke's (2021) recommendations, multiple coders were used to generate a deeper understanding of the data instead of as a measure of agreement. The discussions routinely led to consensus among coders, but the second author was ultimately responsible for preparing the report of the findings.

## RESULTS

Five themes were identified through the analysis. The themes illustrated the mixed experiences of therapists using EFT via teletherapy with couples. Whereas some found it similar to deliver EFT through teletherapy, others experienced exhaustion and barriers to working with specific types of clients. Therapists also experienced some EFT steps, stages, and interventions as more difficult via teletherapy than others and described making adaptations to the model.

### Delivering EFT via Teletherapy is similar to in‐person therapy

About half of the therapists described the delivery of EFT through video‐conferencing as similar to delivering EFT in person. Therapists explained that the formation and maintenance of the therapeutic alliance, as well as the main tasks of stage two of EFT (i.e., pursuer softening and withdrawer re‐engagement) felt similar when delivered through video‐conferencing compared to in person. One therapist said, “I have been able to do some good withdrawer re‐engagement over teletherapy. I think it was as effective as in person. I longed to be in person more during these sessions as it's such a felt experience, but I still felt very moved and the couples did too.” Regarding pursuer softening events, one therapist said, “[There's] no significant difference noted as long as the pursuer is feeling held, heard, and understood by the therapist.”

Therapists also perceived their treatment as having a similar level of success regarding clinical outcomes as services provided in person. Therapists commonly commented, “I haven't noticed too many differences” or “I find little difference.” Another therapist said, “In video sessions, it plays out very much the same. It's EFT in action.” Therapists perceived the delivery and effectiveness of EFT online and in person as similar throughout most of the treatment process, except when developing therapeutic safety, de‐escalating the interactional cycle, and working with highly escalated couples.

The therapists' rate of success with online EFT delivery seemed to surprise them. One therapist explained, “I have been surprised by how effective EFT has been on video. I am able to help clients both ways.” Another therapist said, “I was pleasantly surprised. I wasn't sure I could achieve the same results, but it's been successful.” For many therapists, this sentiment reflected existing and new clients. One therapist commented, “I've even started with new clients and have achieved the same alliance with people I've never met in person with those I have [worked with in person].” These views did not extend to phone sessions; therapists described delivering EFT by phone as being more difficult.

### Delivering EFT via Teletherapy is more challenging than in‐person therapy

About half of the therapists described the use of EFT through video‐conferencing as more challenging than in‐person delivery, while nearly all therapists who delivered EFT by phone felt it was more challenging and less effective. Specifically, intervening over the course of therapy in specific steps of EFT and using EFT with specific clients via video‐conferencing was cited as more difficult than when conducting in‐person sessions. For example, one therapist reported that it took longer to build an alliance over teletherapy saying, “There is a barrier with the screen and being in a different location.…those [clients] that only do telehealth struggle more to allow me to take them to that new, scary place.” Another therapist said, “I find it more difficult to assess the alliance in video. Usually after the initial session, I know that I have made an alliance. In person, it is clearer to me in the moment if there has been misattunement and I can attend to that immediately. There is a distance in video that is not there in person.”

The use of body movement and language were referenced as being a marked difference in EFT delivered via teletherapy. One therapist explained:

I use physical proximity [in person] by leaning in, moving my chair closer, hovering my hand over their space, and standing up or sitting down to communicate connection. I stopped using this online due to the inability to do so. I had to shift to repeating more phrases like: "I hear you. I understand your position. That makes sense. Let me slow you down. What you are saying matters so much. Let's make sure I am getting it.”

Other therapists shared challenges related to creating safety in session via teletherapy. They described concerns with privacy and confidentiality, as well as challenges related to maintaining a safe environment where partners weren't escalated and highly critical of one another. One therapist said, “It's important to me that the client feels safe in their environment when they are in session with me. Sometimes I have to help them find a better place or wait a bit longer for them to get settled before we begin.” Some therapists reported that pursuer softening was particularly more difficult via video‐conferencing. For example, one therapist said, “My pursuers are more escalated during this time period…so it takes more time and more effort to soften them. [They] are more angry and more agitated.…which manifests in their relationship, and they want faster change from their partners.” Fewer therapists described feeling challenged by engaging withdrawers via teletherapy. Other therapists described specific interventions, especially enactments, as more challenging using teletherapy. One therapist explained:

From my experience, it is more difficult online to feel the energy in the room when you are not in the same room. I have missed a few cues and felt more lost online than when I am in the office. Enactments are just as effective but I am more likely to miss something and this puts me at a disadvantage in the fifth step of the TANGO when I try to help them consolidate and make sense of what just happened.

Additionally, some therapists reported their own enjoyment of their clinical work also decreased when using teletherapy, as being online was more draining and exhausting than working in person. One therapist said, “I don't enjoy it. I find it more tiring and less rewarding. My back hurts at the end of the day from leaning forward. My eyes are tired. I miss being able to sit near my clients and touch them during poignant moments.” The lack of enjoyment and burnout were also evident in the remarks of one therapist who said, “The work does not feel as rewarding at a distance. There is something about being in the room as change happens that is missing for me as a therapist. As a result of this and other things, I feel more burnt out than I ever have in my 35+ year career.”

### Delivering EFT via teletherapy is more challenging with certain clients

Therapists described challenges in treating specific types of clients with EFT through teletherapy. The types of clients therapists experienced the most difficulty using EFT through teletherapy with included people with post‐traumatic stress disorder (PTSD), significant trauma histories, high conflict behaviors, and more antagonistic pursuing behaviors. One therapist explained:

The biggest challenge lies more with my dual diagnosis clients, trauma clients, and PTSD clients. It feels easier for me to connect with my clients face‐to‐face, easier to slow the process down and create a corrective experience. I have successfully done this more with clients that I had a strong therapeutic relationship with online and in the office. It is difficult either way when they go back into their trauma and lose safety at home for whatever reason and go back into their pattern. They then bring this to the therapy. I just find it harder to slow them down online. The lag [during video conferencing] disrupts the process. They do slow down. It just feels more abrupt like an interruption rather than a genuine ‘I am here, I am with you’ that the voice and presence carries. They feel it more when face‐to‐face.

Working with couples experiencing high conflict and more hostile pursuing behaviors was more difficult due to not being in a shared space and because the technology didn't allow therapists to speak over and interrupt clients in a way that felt more empathic and calming. One therapist explained, “It's harder to intervene quickly with escalation (e.g., catch bullets) because of the sound delay [during video‐conferencing]. If they are talking, my mic gets cut off and they can't hear me talking to them.” Another therapist said:

It is much harder to contain escalation in video sessions. When couples yell at each other, they can't hear my attempts to calm them down and get their attention because of the way the video software handles audio. In person, I could stand up, put a hand out, roll my chair closer, or in many nonverbal ways contain them. They would also be more likely to hear me saying their names.

### Therapists adapted EFT for Teletherapy

Therapists described ways that they adapted their delivery of EFT though teletherapy. Adaptations included conducting more in‐depth assessment, implementing new safety protocols, asking clients to label their emotions more verbally, containing emotion more, slowing down the process, and incorporating psychoeducation. One therapist described “talking more about the cycle” and “educating” their clients more in video sessions. This was specifically used with highly escalated couples. For example, another therapist reported “I have to do more explaining [about] why I am interrupting them, what process I will be using, getting their buy in and permission to name that I am taking control of the session to stop the escalation.” Another therapist said, “I rely more on explaining and less on client experiencing when I'm [practicing online].” This demonstrated a shift in the more experiential nature of EFT when practiced in person. Additionally, therapists discussed a need to slow down the therapy process to check in with clients more frequently. One therapist said, “I am more aware with online sessions how necessary it is to check with my clients more frequently about how they are reacting to/perceiving what is happening in therapy.”

### Teletherapy and the COVID‐19 pandemic changed therapy practice

During the worldwide COVID‐19 pandemic, therapists were faced with navigating new topics in the therapy room. Many therapists described their clients as being overwhelmed by politics, racial injustices, fears of illness, differences in risk tolerance regarding COVID‐19 safety measures, and navigating being locked down together. Therapists also described experiencing an abundance of discussion on parenting, homeschooling, isolation, loneliness, mood disorders, substance use, and anxiety. For example, one therapist noted that all their clients had been impacted by the COVID‐19 pandemic, “They are all struggling with depression, anxiety, [and] substance abuse in much higher levels. Without the support of time with friends and family members, and being trapped only with their partners, people are more irritable…I've never seen anything as stressful as this period.” Another therapist stated, “Most speak about how hard it is to manage the schooling of their children on top of everything else. They also speak about how their children are struggling psychologically.”

Therapists explained that the transition to teletherapy altered their future plans for their practice. While some therapists planned to only offer in‐person sessions after the pandemic restrictions were lifted, many therapists described plans for a hybrid approach or only offering teletherapy in the future. One therapist reported enjoying providing teletherapy; they had “closed one office” and “will likely close the second office” to only provide online sessions. Another therapist said, “I think being in my own environment, having my own attachment figures in my day, love from my dogs, better eating, less commuting, sleeping more, helps me show up with more energy and more focus. I will always keep telehealth as my more dominate form of service.”

## DISCUSSION

This study examined the teletherapy experiences of certified EFT therapists who transitioned to teletherapy during the COVID‐19 pandemic. Overall, the results provided support that EFT therapists can deliver EFT online. Interestingly, although therapists indicated EFT online is similar to in‐person therapy, therapists also reported unique challenges, including de‐escalating high conflict relationships and ensuring physical and emotional safety among partners. Therapists also reported making adaptations when delivering EFT through teletherapy, such as a more rigorous screening for safety and providing more psychoeducation about a wide range of topics including relational cycles and EFT.

The results of this study align with findings from both the survey research by Hardy et al. (2021) and the study by Heiden‐Rootes et al. (2021). Hardy et al. (2021) found that most couple and family therapists were able to successfully conduct couple therapy online; however, it was more challenging to re‐direct highly escalated couples, harder to create therapeutic presence, more difficult to create safety, and necessitated a slower pace. Although the therapists in the Hardy et al. (2021) and Heiden‐Rootes et al. (2021) studies did not discuss increasing the level of psychoeducation they provided to clients, they did discuss increasing structure, being more directive, and slowing therapy down, all of which are echoed in the findings from the present study. Therapists in this study also noted increased fatigue, although most of them planned to continue providing teletherapy after restrictions were lifted (Hardy et al., 2021; Heiden‐Rootes et al., 2021).

Some therapists hold negative views of teletherapy and may be skeptical of the ability to engender therapeutic presence online (Geller, 2021; Rees & Stone, 2005), yet teletherapy increases accessibility (Connolly et al., 2020; Glass & Bickler, 2021), some clients may favor teletherapy (Maier et al., 2021), and research indicates therapeutic alliance can be successfully created online (Maier et al., 2021; Simpson & Reid, 2014). CFTs have been slower to embrace teletherapy (Hardy et al., 2021), perhaps because of the challenges of creating therapeutic alliance online with multiple people (Wagnild et al., 2006). Some therapists in the present study indicated that they were surprised that they could create an equally strong therapeutic alliance online compared to in person, despite prior findings indicating that a teletherapeutic alliance can be equally as strong as an alliance built in person (Davis et al., 2023).

### Practice recommendations

Although many EFT therapists reported working online was more challenging than conducting in‐person sessions, they provided key strategies that are useful for EFT therapists who plan to use teletherapy. We have integrated participants' recommendations and resulting themes with key recommendations from the literature to offer practice recommendations for delivering EFT via teletherapy. Many recommendations can be extended more broadly to all evidence‐based couple therapy delivered through teletherapy.

#### Preparation

Geller (2021), Bennett (2020), and Jorgensen and Gould (2020) discuss the need to intentionally prepare to work online. For example, planning for technology glitches and receiving specific training for providing teletherapy can significantly improve therapists' abilities to strengthen the teletherapeutic relationship (Geller, 2021; Pierce et al., 2020). Indeed, training in teletherapy appears to increase therapist competency and perceived effectiveness (Dopp et al., 2021). Additionally, to ensure therapeutic skills are maintained and ethical codes are followed, therapists should receive supervision on teletherapy (Bernhard & Camins, 2020). This recommendation integrates both the first and second themes generated in this research, as both training and supervision are essential for CFTs seeking to develop new skills, use new modalities, and navigate challenges and changes in their clinical practice (Jordan, 2015).

Bennett (2020) suggests focusing more on planning sessions, stabilization, and ensuring therapist and client privacy. This recommendation aligns with the third and the fourth themes, as EFT therapists noted an additional need for planning, stabilization, and increased privacy when working online. To do so, therapists should create a welcoming, distraction‐free, and consistent space in which to do teletherapy (Geller, 2021; Jorgensen & Gould, 2020). Geller (2021) also suggests that maximizing therapist safety and presence involves having high‐quality lighting and the camera at eye level as well as sitting a safe and comfortable distance from the screen. If a change in location is required, Bennett (2020) recommends taking time to prepare clients for new spaces.

Heiden‐Rootes et al. (2021) and Geller (2021) report teletherapy engenders both therapist creativity and exhaustion, indicating therapists may need to structure their sessions in ways that allow for rest. Indeed, extant research indicates people who use video‐conferencing platforms for extended periods of time report fatigue, anxiety, distraction, and apathy as well as difficulty focusing and remembering (Bennett et al., 2021; Bothra, 2020). To navigate this fatigue, EFT therapists may consider structuring sessions earlier in the day (Bennett et al., 2021), integrating standing and stretching breaks between sessions, and attending to their ocular health by focusing on more distant objects as frequently as is feasible (Ramachandran, 2021). Additionally, EFT therapists want to ensure their telehealth platform settings allow for maximum comfort (Peper et al., 2021), reduce on‐screen stimuli (Ramachandran, 2021; Wicks, 2021), hide their ‘self‐view’ to reduce excess stimuli (Ramachandran, 2021; Wicks, 2021), and use bodily movements to stay engaged (Peper, 2021; Ramachandran, 2021).

Therapists should also prepare both themselves and their clients to effectively transition in and out of sessions. Geller (2021) recommends the therapist take 5–10 min prior to each session to engage in a mindfulness practice. Engaging in a presence‐focused practice has been shown to positively impact therapeutic alliance (Dunn et al., 2013). Because of the strain of teletherapy, therapists may need more time for self‐care between sessions (Geller, 2021). This may indicate therapists need to schedule fewer sessions per day and/or schedule sessions with breaks in between. Additionally, Geller (2021) notes it is important to create a ritual for transitioning out of the teletherapy space at the end of the day by moving, stretching, and closing the virtual office space. For clients, Brenner (2021) notes the importance of taking more time to both open and close a session, especially one that has been more escalated and/or involved more intense emotional experiences. Brenner (2021) recommends giving clients additional time to settle in and asking clients about their intentions for the session. Bennett (2020) and Jorgensen and Gould (2020) note it is important to mindfully end a session, especially if clients are returning to spaces that can be emotionally challenging. Therefore, therapists may want to start to prepare clients for ending the session earlier than they would in person and help clients name how they will care for themselves after the session has ended.

#### Assessment

When using teletherapy, EFT therapists reported modifying the assessment strategies outlined by Johnson (2019) to ensure client safety and privacy, which is reflected in the third and fourth themes. Risk assessment and safety intervention can successfully occur via teletherapy (Myers et al., 2017). When conducting risk assessment, previous recommendations include assessing for harm to self (Holland et al., 2021), determining the physical location of the client (Holland et al., 2021), ensuring the client has sufficient privacy (Jorgensen & Gould, 2020), and assessing partners separately (Wrape & McGinn, 2018). Privacy can be encouraged by asking clients to wear headphones, especially if intimate partner violence (IPV) is suspected (Wrape & McGinn, 2018).

Aligned with the findings in this study (i.e., theme three), Hogan (2022) reports that the most significant difference in teletherapy is the ability to control the therapy room. Therefore, screening for safety (e.g., IPV) and any issues that may impede communication (e.g., hearing difficulties) ensures clients have the resources they need for teletherapy (Hogan, 2022; O'Reilly Treter et al., 2021). Burgoyne and Cohn (2020) expand on this by providing an assessment checklist for teletherapy that includes the client's cognitive status, their technological and physical space resources, and any potential safety risks. Participants in this study also expanded on these previous recommendations by discussing the need to conduct more stringent assessment by explicitly asking about client physical, emotional, and financial safety as well as suicidal ideation. EFT therapists also noted assessing extra‐therapeutic resources more often, including familial relationships and financial supports after job loss. Using teletherapy, EFT therapists may need to go beyond the usual EFT assessment plan to assess for current macrosystemic influences on client intrapersonal and interpersonal health.

#### Implementing new safety protocols

In addition to assessing for safety more frequently (i.e. theme four), EFT therapists noted a need to implement new ways of creating safety with clients, such as conducting sessions at times that ensure privacy, being flexible about session location, and ensuring that the location where the therapist was conducting sessions was familiar to the clients. Jorgensen and Gould (2020) discuss the need to create a familiar and intentional therapeutic space for clients within the teletherapy screen. Maintaining rituals and routines is helpful during times of upheaval and stress (Jorgensen & Gould, 2020) and this can include the therapist continuing to work from their office and/or scheduling clients at the same time of day when transitioning online (Jorgensen & Gould, 2020). Therapists should also confirm they have the client's most recent contact information to ensure client safety or in the event of a disconnection (Jorgensen & Gould, 2020). Therapists cited disruptions due to other household members, such as children, as a primary privacy concern. Jorgensen and Gould (2020) and Wrape and McGinn (2018) recommend the use of headphones to increase client privacy.

While therapists can never completely ensure client privacy when working online (Burgoyne & Cohn, 2020), steps can be taken to create safety within a private space, such as more stringent assessment of which types of clients are appropriate for teletherapy, informed consent (Wrape & McGinn, 2018), and directly discussing the nature of doing therapy online (Jorgensen & Gould, 2020). Additionally, when working with high conflict couples, it may be necessary to work with each partner separately for a time prior to conducting conjoint sessions (Hogan, 2022), implement turn taking (Burgoyne & Cohn, 2020), encourage clients to only talk to the therapist, not their partner, (Allan et al., 2020), and/or use separate rooms and screens (Burgoyne & Cohn, 2020).

Furthermore, as recommended per Hoss et al. (2023) and Tseng et al. (2022), EFT therapists should also assess for technology abuse [i.e., the use of technology to perpetuate harm, violence, and/or control to instill fear (Markwick et al., 2019)] as part of IPV assessment. For example, therapists should assess clients' experience of technological abnormalities (such as installed applications that clients do not recognize), whether the client's location seems to be consistently tracked by their partner, and the degree to which clients experience incessant calls and/or messages from their partners (Hoss et al., 2023).

#### Making emotions explicit

Geller (2021) notes the challenges of developing and maintaining therapeutic presence and alliance when working online. For example, the development of a trusting, safe, co‐regulatory therapeutic relationship is challenging because of the perceptual limitations of non‐verbal communication (Bos et al., 2002; Geller, 2021; Oshni Alvandi, 2019). Indeed, non‐verbal communication makes up to 70% of the social meaning during a conversation (Birdwhistell, 1970) and this can be significantly limited online because of the space and size of screens. To account for this, Geller (2021) and Glass and Bickler (2021) recommend teletherapists focus on their facial expressions, pace and tone of voice, mutual eye gaze, non‐verbal gestures, and mirroring clients' expressions and breathing.

The therapists in this study emphasized how more subtle emotions and micro‐expressions were harder to assess via teletherapy (i.e., theme two), echoing the findings by Burgoyne and Cohn (2020). Therapists, therefore, needed to ask directly and more explicitly about client emotional experiencing. Bennett (2020) recommends therapists using teletherapy ask questions that seem ‘overly obvious’ about emotions to validate that the therapist is engaged with the client. Therapist vulnerability models for clients how to engage emotionally online (Jorgensen & Gould, 2020); bringing attention to more subtle emotions and checking in with clients more (i.e., theme four) ensures EFT therapists are accurately tracking and reflecting their clients' experiences (Jorgensen & Gould, 2020) and drawing clients' attention to their own emotional experiencing (Burgoyne & Cohn, 2020).

#### Slowing down

EFT therapists in this study frequently stated the need to slow down when using teletherapy (i.e., theme four). Bennett (2020) echoes this need, reporting that the lack of physical presence prevents therapists from using nonverbal cues to influence the clients during session. Therapists, therefore, may need to interrupt and ask clients to slow down more frequently (Bennett, 2020). This could be done to hold on to an intense moment, deepen awareness of intrapersonal experience, or listen more closely to therapist suggestions (Bennett, 2020). Therapists working via telehealth may need to ask about a client's facial expressions, body language, or perceived affect more explicitly, especially if there are lighting or technology concerns (Wrape & McGinn, 2018).

Therapists also reported EFT teletherapy taking longer (i.e., themes two and four): building rapport, cycle de‐escalation, and accessing primary emotions were all reported to take longer online. Research is mixed regarding the depth and length of time it takes to establish a strong therapeutic alliance via teletherapy (Greene et al., 2010; Morland et al., 2015). Therefore, EFT therapists should be prepared to intentionally focus on rapport building with teletherapy clients for a longer amount of time than when working in person. Therapists should also be prepared to repeat themselves more often (Jorgensen & Gould, 2020) and take more time accessing primary emotions while de‐escalating the negative cycle.

EFT therapists frequently use non‐verbal communication to convey their therapeutic presence (Johnson, 2019). For example, during pursuer softening and withdrawer re‐engagement, therapists often use their gaze or physical gestures to soothe the partner who is not the focus of the intervention. As evidenced by the second theme, this can be significantly more challenging online, as both therapists and clients may miss each other's micro‐expressions and non‐verbal communication (Geller, 2021). As such, therapists may want to shift how they are narrating the session. For example, Brenner (2021) describes increasing the level of transparency in the therapeutic relationship. Specifically, Brenner (2021) describes naming their felt sense more often, explaining their therapeutic intentions, making the implicit more explicit, interrupting more often, and obtaining more client feedback. In stage two of EFT, this may look like the therapist explaining their intention of accessing core attachment fears and longings to increase emotional coherence and responsiveness and routinely checking in with both partners to ensure they feel supported by the therapist.

#### Psychoeducation

Finally, EFT therapists found themselves providing more psychoeducation via teletherapy (i.e., theme four). This included explicitly discussing how safety is created, the nature of teletherapy, and the actions taken by the therapist. Jorgensen and Gould (2020) discussed the need to be more explicit about therapist behaviors with clients. Clients also benefit from explicit discussions about teletherapy, therapeutic limits when working online, and what client behaviors are appropriate during session (Jorgensen & Gould, 2020; Wrape & McGinn, 2018). Wrape and McGinn (2018) describe the need for therapists to have clients practice skills both in session and at home to ensure clients understand the therapists' directives. EFT therapists may find themselves initially working in a more cognitive or behavior‐based way while establishing rapport and connection prior to deepening client emotional experiencing when using teletherapy.

## LIMITATIONS AND FUTURE RESEARCH

The study offered a breadth of insights about the use of teletherapy for EFT practice with couples. However, one significant limitation of qualitative surveys is the inability of researchers to probe for understanding. Responses tended to be brief and future research could utilize our findings to design a more in‐depth and mixed method inquiry for understanding EFT delivered via teletherapy. The length of qualitative surveys can also be challenging: several participants in the study noted that the study was long or that they had already answered a question in a previous response. Additionally, our sample was limited by the racial and gender identities with largely White and female perspectives. Recruitment focused on EFT clinicians who transitioned to teletherapy during the stay‐at‐home orders in the COVID pandemic. Findings may have been limited since therapists may have had limited prior teletherapy experience before the pandemic. Finally, as with all qualitative research, generalizability is limited. Braun and Clarke (2006, 2021) position generalizability as the ability of a study to apply across populations; while this study may (generally) reflect the experiences of White, female, EFT therapists, it may not fully reflect all therapists' experiences of delivering EFT via teletherapy. Therapists in this study described adapting their delivery of EFT for teletherapy. Systematic research that identifies adaptations, translates EFT for online delivery, and tests the efficacy and effectiveness of EFT delivered via teletherapy is needed. Since behavioral approaches are thought to translate more easily to online delivery compared to experiential interventions like EFT, it may be useful to test the efficacy of online EFT versus an online behavioral approach. Given the scarce existing research on teletherapy for couples combined with the increase in the utilization of teletherapy, this research should be a high priority to ensure the effectiveness and safety of EFT delivered via teletherapy.

## CONCLUSION

This paper describes the experiences of EFT therapists using teletherapy and, subsequently, generates recommendations for EFT practice via teletherapy. This study presents several findings, including that delivering EFT online may present challenges, adaptations may need to be made, and some relationships may be more challenging to work with online or may not be appropriate for teletherapy. Future research should explore adaptations for delivering EFT online, the efficacy of EFT online, and the impact of limited non‐verbal communication on the therapeutic alliance and depth of emotional experiencing in EFT.
